# Point-Cloud Instance Segmentation for Spinning Laser Sensors

**DOI:** 10.3390/jimaging10120325

**Published:** 2024-12-17

**Authors:** Alvaro Casado-Coscolla, Carlos Sanchez-Belenguer, Erik Wolfart, Vitor Sequeira

**Affiliations:** 1 European Commission, Joint Research Centre (JRC), Via Enrico Fermi 2749, 21027 Ispra, Italy; alvaro.casado-coscolla@ec.europa.eu (A.C.-C.); erik.wolfart@ec.europa.eu (E.W.); vitor.sequeira@ec.europa.eu (V.S.); 2 Instituto Universitario de Automática e Informática Industrial, Universitat Politècnica de València, Camí de Vera, 46022 València, Spain

**Keywords:** 3D data mining, 3D instance segmentation, LiDAR, deep learning

## Abstract

In this paper, we face the point-cloud segmentation problem for spinning laser sensors from a deep-learning (DL) perspective. Since the sensors natively provide their measurements in a 2D grid, we directly use state-of-the-art models designed for visual information for the segmentation task and then exploit the range information to ensure 3D accuracy. This allows us to effectively address the main challenges of applying DL techniques to point clouds, i.e., lack of structure and increased dimensionality. To the best of our knowledge, this is the first work that faces the 3D segmentation problem from a 2D perspective without explicitly re-projecting 3D point clouds. Moreover, our approach exploits multiple channels available in modern sensors, i.e., range, reflectivity, and ambient illumination. We also introduce a novel data-mining pipeline that enables the annotation of 3D scans without human intervention. Together with this paper, we present a new public dataset with all the data collected for training and evaluating our approach, where point clouds preserve their native sensor structure and where every single measurement contains range, reflectivity, and ambient information, together with its associated 3D point. As experimental results show, our approach achieves state-of-the-art results both in terms of performance and inference time. Additionally, we provide a novel ablation test that analyses the individual and combined contributions of the different channels provided by modern laser sensors.

## 1. Introduction

Object recognition plays a central role in many automated systems, like autonomous driving, mobile robotics, surveillance applications, and many others. It mainly involves three major tasks: detection, classification, and segmentation, and it is typically applied to RGB camera images. Cameras have the advantage of offering a good balance between price and performance, providing high-resolution images with no range limitations. Laser sensors, on the other hand, have been traditionally excluded from these tasks due to their higher prices, bulkiness, and limitations in terms of range, field of view, spatial/temporal resolution, and data sparsity. However, over recent years, the rise of the autonomous driving sector has fostered their development, addressing most of their major drawbacks and, consequently, reducing the gap between cameras and laser sensors.

When compared to cameras, laser sensors have three main advantages for object recognition: (1) they are inherently more robust, as they are not influenced by changes in light and low-visibility environments; (2) they deliver consistent depth information; and (3) background subtraction operations are simpler. As with cameras, spinning laser sensors produce structured data: all measurements can be mapped into a 2D grid without loss of information. The position of an element inside the grid, together with the projection model of the sensor, allows the calculation of its associated view ray. Also, as cameras do, the locality of measurements is preserved, i.e., two contiguous points in the 3D Cartesian space remain contiguous in the 2D projective space.

Convolutional Neural Networks (CNNs) benefit from this locality to learn features that allow performing object recognition tasks. DL techniques have achieved a significant breakthrough in this field and are, de facto, the preferred approach for addressing these kinds of problems. A wide variety of models is publicly available, and a state of maturity has been achieved for analyzing 2D visual information. However, when it comes to 3D data, the degree of maturity is significantly lower, with less literature and considerably lower performance, both in terms of robustness and computational requirements.

### 1.1. Related Works

Traditionally, 3D object recognition was based on hand-crafted methods. The intrinsic complexity of the different tasks involved, together with the simplifications of such methods, imposed a hard limit on the overall performance, generality, and robustness. During the last decade, with the rise of deep learning and the improvements in 3D sensors (both in terms of quality and availability), a vast number of works have been published in this field. An exhaustive survey on such techniques can be found in [1,2,3] and the references therein.

One proof of such an explosion is the ever-growing number of publicly available annotated datasets that can be used for training this kind of model. Some of them focus on classification [4,5,6,7], some on detection [8,9] and others on segmentation [10,11,12].

Literature on 3D object recognition has a clear focus on the general problem: unstructured point clouds (i.e., point clouds in which points are randomly ordered, not implicitly encoding neighborhood relationships). Paradoxically, many relevant applications rely on real-time structured sensors, like spinning lasers, e.g., autonomous driving or mobile robotics. Alternatively, some applications [13] make use of RGB-D cameras that output range maps in addition to RGB images. The structured nature of range maps makes it possible to apply well-known 2D convolutional architectures. However, these sensors offer lower accuracy and limited range when compared to LIDAR sensors. In addition, they are more sensitive to changes in lighting conditions.

Unlike images, general point clouds lack structure. This, together with the increase of dimensionality in the data, poses a great challenge when facing the perception problem and explains the gap in performance between state-of-the-art techniques in the 2D and 3D domains. Current techniques work either over raw points, discretized versions of the point clouds (volumetric methods), or projections. The choice of representation plays a critical role in the final performance and imposes certain architectures for the underlying models.

Point-based methods work over unordered and unstructured data, which makes it unfeasible to directly apply standard convolutional operators. To capture inherent per-point context features, the most common approaches rely on MultiLayer Perceptron architectures [14,15,16,17,18], Recurrent Neural Networks [19,20] or graph representations [21,22]. Alternatively, other techniques propose point convolution operators that exploit the vicinity of points in the Euclidean space [23,24,25]. These kinds of techniques are lossless, i.e., they work over the raw data but are limited due to the lack of structure. This has an impact in terms of computational cost and makes the learning problem more complex.

Volumetric methods work over a spatial discretization of the original point cloud. They overcome the lack of order and structure in the data by re-sampling it. This allows for simpler 3D convolutions at the expense of losing some information and introducing discretization artifacts. Depending on the way the point cloud is discretized, it can be distinguished between dense [26,27,28] and sparse [29,30,31] techniques. The first suffers more from memory requirements, while the second may introduce a computational overhead.

Projective methods address the main issues of 3D data by working over 2D views of the point cloud. This enables the use of well-known 2D models but comes with the cost of losing information. Typical projections include Bird’s-Eye-View [32,33], multi-view planar projections [34], spherical projections [35] or cylindrical projections [36].

A common alternative to the former techniques consists of fusing laser sensors and cameras. Recent works [37,38] have studied the fusion of point cloud and image features using advanced architectures, like transformers, achieving state-of-the-art performance in 3D object detection. Nevertheless, perception tasks are often performed over the images, and the results are projected into the point cloud [39,40,41,42]. This enables the use of state-of-the-art 2D object recognition models over visual information like YOLO [43], RetinaNet [44], or SSD [45] and to benefit from the range measurements of laser sensors. This kind of approach is particularly popular in real-time systems, where pure 3D methods are not fast/reliable enough.

If the scope of the problem is further constrained and, instead of working over general point clouds, the focus narrows down to structured ones, the number of published works is considerably reduced. This is probably due to the fact that, traditionally, spinning laser sensors had a very poor vertical resolution and, consequently, the amount of data available was insufficient. However, considering that current sensors provide up to 128 lines, this is no longer the case.

The first work to identify this potential was [46], where the authors treated the structured data as a set of images to build a saliency map that could be used for tasks like obstacle detection. Then, in [47], it was demonstrated that these kinds of images contained enough information for some self-driving tasks and showed their invariancy to seasonal changes and environmental conditions.

The only work addressing the perception problem exclusively from structured point clouds is [48]. Here, the authors highlighted the feasibility of using spinning laser sensors as low-resolution cameras and evaluated the performance of different 2D object detectors over LiDAR images. For such evaluation, the images were adapted to the input format of the object detectors, but no specific training was performed to fine-tune the models for this new type of input.

### 1.2. Overview

In this work we benefit from the state of maturity of 2D visual models to directly apply them into the 3D domain. To do so, we exploit the structured nature of spinning laser sensors. The main contributions of this work are:

- We cast the 3D instance segmentation problem for structured spinning lasers to the 2D domain without explicitly re-projecting the 3D data. By relying on the implicit structure imposed by the electromechanical design of the sensors, we achieve a lossless mapping that preserves data locality and saves computational resources. To the best of our knowledge, this is the first approach that does not rely on a projective model for applying 2D instance segmentation CNNs, e.g., spherical or cylindrical projective techniques.
- We exploit simultaneously all the channels that modern laser sensors provide, i.e., range, reflectivity, and ambient light, which offer complementary information that can be exploited for object recognition tasks.
- We introduce a novel, general-purpose, 3D data-mining technique that allows the automatic annotation of point clouds without human intervention under controlled circumstances. Together with this paper, we present a new public dataset with all the data collected for training and evaluating our approach, where point clouds preserve their native sensor structure and where every single measurement contains range, reflectivity, and ambient information, together with its associated 3D point.
- In the results section, we provide the results of a novel ablation test that analyses the individual and combined contributions of the different channels provided by modern laser sensors.

The remainder of this document is structured as follows: Section 2 gives a detailed explanation of our approach, distinguishing between the data-mining technique and the inference pipeline. Section 3 presents our results by performing a detailed ablation test and compares our technique with other state-of-the-art models. It also provides details on the public dataset that accompanies this paper. Finally, Section 3 outlines the main conclusions and future works.

## 2. Materials and Methods

In this paper, we focus on using a specific spinning laser sensor, an Ouster OS0-128 (Ouster’s website: https://ouster.com accessed on 10 December 2024), and a specific CNN architecture, a YOLOv8 CNN [49] with segmentation capabilities. Even though this choice imposes some implementation constraints, the fundamental ideas presented here remain generic and can be extended to different devices/models.

The Ouster OS0-128 sensor acquires real-time point clouds with a vertical field of view of 90 degrees and a horizontal field of view of 360 degrees. It provides a fixed height of 128 rows and a configurable width that ranges between 512 and 2048 columns. For our approach we configured it to work with 1024 columns, as it gives a reasonable resolution ratio and allows us to better integrate the data with the YOLO standard input size. For every point measured, we consider three channels: its distance to the sensor (*range*), its calibrated *reflectivity*, which accounts for the number of laser photons that the surface of the object reflects after compensating for distance and sensitivity of the detector, and the near-infrared *ambient* light that it reflects.

**Figure 1:**
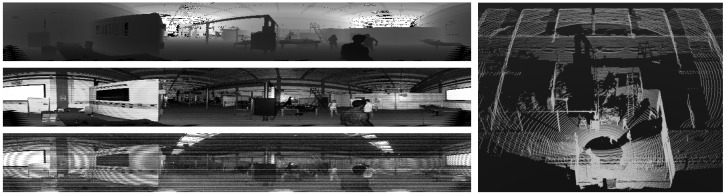
Ouster data used in this paper. (**left**) Structured view in the projective space (1024 × 128 pixels). From top to bottom: range, reflectivity, and ambient channels. (**right**) Partial view of the associated point cloud in the 3D Cartesian space.

Figure 1 shows a sample scan of the Ouster OS0-128 sensor with all the data we use in this paper. Notice how the three channels considered offer complementary information that can be exploited for object recognition tasks: range information defines sharp edges around objects, while the reflectivity and ambient channels provide textural information under two different types of illumination.

On the other hand, the YOLOv8 CNN takes as input images with a size of 640 × 640 × 3, where the three channels were originally intended for RGB intensities. For each detection, it returns a 2D bounding box, a confidence value, a label, and a segmentation mask.

### 2.1. Data Mining

Our data-mining technique relies on a background removal strategy that benefits from the robustness of range measurements and the light invariance provided by laser sensors. It is based on the assumption that training data can be acquired under controlled circumstances. In this sense, we impose three constraints: (1) the sensor is mounted on a static position, (2) the environment can be acquired *empty*, i.e., with no objects of interest in it, beforehand and remains static, (3) only instances of a single class are visible to the sensor during each acquisition. Such conditions may be excessively limiting for specific use cases, e.g., autonomous driving, but are trivial to achieve in many other applications, like surveillance or process monitoring.

The first two conditions allow the building of a 3D model of the background: first, we accumulate all the points acquired by the sensor during a short period of time. Then, we define a voxel structure where all cells containing these points are flagged as *full*. From this moment, all new points reported by the sensor that fall into full voxels are considered to be the background and the rest as the foreground.

The main advantage of this approach is that background/foreground segmentation becomes very efficient in terms of computational complexity and that it copes very well with sensor noise: since the background model is based on several consecutive scans, it also contains most of the artifacts that the sensor introduces due to mechanical or optical limitations (like flickering edges).

The third constraint allows the labeling of all the foreground points as the class that is being acquired. To do so, and to account for situations in which multiple objects of the same class are present in a single scan, a clustering operation becomes necessary. Such operation is not only necessary for separating instances, but also for removing the noise that the background model could not filter out.

**Figure 2:**
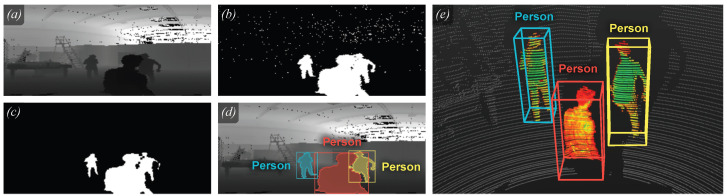
Background removal and foreground clustering. (**a**) Partial slice of a scan (range channel). (**b**) Segmentation mask after voxel filtering. (**c**) Resulting mask after the *shrink* operation (seeds for the flooding algorithm). (**d**) Masks of the resulting clusters, with their associated labels. (**e**) 3D view of the clusters with their 3D bounding boxes.

Our technique performs the clustering operation with a flooding algorithm. Figure 2 illustrates the complete process. Given a set of foreground points, P, (Figure 2b) and a set of seeds, S⊂P, (Figure 2c), we start with a random seed, $s_{i}\in S$, and collect all the points from P that are *reachable* from $s_{i}$. This process is repeated until all points in S have been visited, producing at each iteration a new cluster (Figure 2d). We consider a point to be reachable from seed as long as they can be connected between each other by following a sequence of *neighbor* points. Two points are considered to be neighbors if they are contiguous in the projective space and their associated Euclidean distance is below a given threshold (that compensates for data sparsity by considering the distance).

We compute S by performing a binary *shrink* operation over P. This morphological shrink operation, also known as erosion, consists of removing a layer of pixels from a foreground region around all its borders against the background, i.e., eroding one pixel along all its boundaries. This distinction allows the acceleration of the algorithm and the effective removal of sensor noise, as some points in P will never be reachable from S. Additionally, it does not degrade the edges of clusters as a binary *shrink* does (notice the differences between contours in Figure 2c,d). Figure 2d,e show how overlapping instances in the projective space can be easily separated using range information (red and yellow persons). This separation with only visual information would be more challenging and could potentially introduce errors.

This technique can be easily generalized to unstructured data: S could be extracted from P with an *shrink* operation over voxels, and the flooding algorithm could traverse the point cloud also with voxels or a 3D radius search.

### 2.2. Inference Pipeline

Even though spinning laser sensors provide structured point clouds, they differ w.r.t. cameras in both the domain of the data and resolution/field of view. Since the goal of this work is to rely on DL models originally intended for visual information without altering their architecture, it becomes necessary to remap the data and retrain the model.

**Figure 3:**
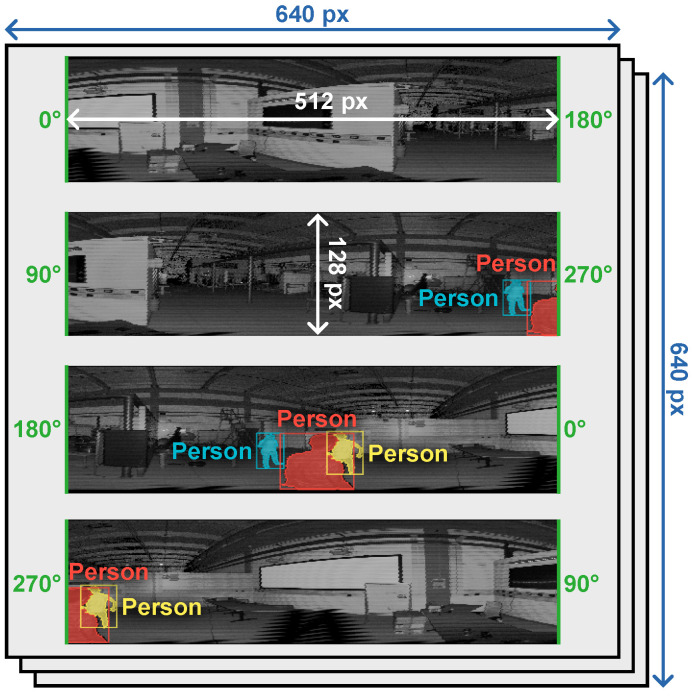
Data remapping from the projective space to the CNN space. All three channels (range, reflectivity, and ambient) are split into four overlapping segments and stacked together to comply with the expected input tensor size (640 × 640 × 3). Segmentation masks and bounding boxes are split accordingly. Only the reflectivity channel is represented in this figure.

To fit the Ouster data into the YOLOv8 standard input (from here on *CNN space*), we stack the three channels provided by the sensor (range, reflectivity, and ambient) into the three input channels originally intended for RGB intensities. From a spatial point of view, each laser channel has a size of 1024 × 128 and needs to be mapped into a 640 × 640 tensor. We overcome this by splitting every channel into four segments of 512 × 128 that cover 180 degrees horizontally each and that overlap 90 degrees with the contiguous ones. From a data point of view, both reflectivity and ambient channels are normalized using a histogram equalization, while for the range channel, we clamp and normalize it using a fixed maximum distance. Figure 3 shows the CNN space for the scan illustrated in Figure 1, where only the reflectivity channel is illustrated.

This representation allows fitting all the data into the CNN space and overcomes potential issues when objects are placed around the $0^{\circ }$ direction from the sensor, e.g., notice the white projector screen split in Figure 1-left. The remapping ensures that any object that covers, at most, $90^{\circ }$ horizontally will be fully visible in one of the segments (see Figure 3 where the segment that covers the $\left[270^{\circ },90^{\circ }\right]$ interval contains the full projector screen).

Segmentation masks and bounding boxes are remapped accordingly for the training. Consequently, single instances are represented more than once in the CNN space. This does not necessarily have a negative impact since a full view is ensured as long as the object covers less than 90 degrees. Instead, it can be beneficial as it indirectly augments the input samples by introducing partial views and different locations for the same instances. Notice from Figure 3 how all the masks shown in Figure 2 produce at least two different detections in the CNN space. Also, notice how the red one, which falls in between two edges in the CNN space, produces three different detections (one complete and two partial views).

When performing inference, the output of the model is expressed in the CNN space. Consequently, the predicted bounding boxes and segmentation masks need to be remapped back to the sensor’s projective space and fused. This is a straightforward process where partial detections, i.e., the ones near the edges of the segments, can be discarded and where a standard non-maximum suppression algorithm can identify overlapping detections. Thanks to our data representation, the risk of missing a detection is mitigated as instances always have a full view in one segment and, potentially, up to two.

**Figure 4:**
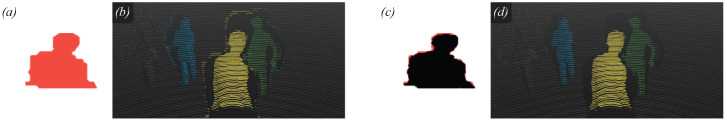
Segmentation masks fusion with range information. (**a**) Raw mask of the yellow person in (**b**), as inferred by the model. (**b**) Direct projection of the raw masks to the 3D data. Notice the artifacts around the edges. (**c**) Largest cluster after the flooding algorithm over a), i.e., the final segmentation mask, with its associated raw mask on the back. (**d**) Results after projecting the final segmentation masks into the 3D data.

The final segmentation masks typically need to be combined with the sensor’s range data for further processing, e.g., some applications may need to compute the 3D bounding boxes of the detections or to track instances. However, such masks are not necessarily consistent with the 3D information: small errors, especially along the edges, may compromise the results (see Figure 4a,b). Such errors may be a consequence of imperfect predictions but, in the case of YOLOv8, are heavily affected by the resolution of the resulting segmentation masks (1/4 of the input image). This imposes an up-sampling operation for mapping the masks into the sensor space and yields a data loss.

We mitigate these artifacts by running the same clustering algorithm as the one detailed in Section 2.1. In this case, P corresponds with the binarized mask predicted by the model (Figure 4a), while S remains as the result of a *shrink* operation over P. Only the points belonging to the largest cluster are kept for the final segmentation mask. The outcome is a subset of points of the initial prediction that is free of discontinuities in the Cartesian space and, thus, where edge artifacts have been removed (see Figure 4c,d).

## 3. Results

To validate our automatic data-mining technique, train the model, and assess our pipeline’s performance, we acquired and labeled our own dataset using an Ouster OS0-128 sensor. We could not rely on already existing datasets as they provide unstructured data, with only range and reflectivity channels (no ambient light) and with lower vertical resolution (64 lines in the best case). Since the main novelty of this work consists of avoiding re-projections to achieve a structured representation and exploiting all three channels simultaneously, using these datasets would prevent us from assessing the benefits of such contributions. All the material used in this paper, including the raw annotated scans and the training and evaluation samples, are publicly available for reproducibility and for future developments and benchmarks.

This section covers three main topics: (1) the evaluation of the data-mining technique, together with a brief description of the public dataset; (2) an ablation test that details the individual contributions of the building blocks of our pipeline; and (3) a quantitative comparison of our technique with other state-of-the-art models for 3D object detection, trained and evaluated with our dataset.

### 3.1. Data Mining

We recorded 12 sequences of two minutes each. We focused on a single-class classification task (persons), and most of the acquisitions were performed in indoor environments. As Table 1 shows, our dataset [50] is composed of 14,364 scans, from which 5.47% (786) contain only background information (second column) and the remainder 94.53% (13,578) contain one or more instances (third column). In total, the dataset contains 28,198 instances (fifth column), and for each one, a binary mask is generated.

All the labeling was automatically performed by our data-mining technique. However, to ensure correctness and to evaluate the robustness of our approach, a visual review was performed to spot incorrectly labeled scans (fourth column). In total, we identified 2.32% (334) scans in which our technique failed.

The main source of such errors was situations in which partial occlusions split a single instance into two different clusters. On rare occasions, some incorrect masks were also produced due to the proximity between two persons or between a person and the background. In the first case, the two persons were considered to be a single instance. In the second, the mask was incomplete (some of the points were considered to be background).

The other limitation is related to the representation of the background: since we considered a voxel size of 15cm, the instance points next to the floor (typically the feet of the persons) are only partially captured by the mask.

Regardless of the minor errors that we identified, and for completeness, we decided to leave in the dataset all the scans. The incorrect ones are flagged as so and carry the (wrong) masks that were produced by our technique. For the training/evaluation, only correct scans were considered.

### 3.2. Inference Pipeline

We performed several experiments to measure how descriptive are the different channels of the laser and what is the impact of using a more complex model. We also analyzed the individual contributions of the CNN and the post-processing pipeline. To do so, we trained 35 different models using 4000 scans randomly selected among all the acquisitions, except for *lab5* and *out1*, which were left aside for evaluation.

For assessing the impact of the different laser channels, we considered all possible combinations of them: only ambient light (A), only depth (D), only reflectivity (R), only two channels (A+D, A+R, D+R), or all of them together (A+D+R). To maintain a homogeneous architecture along the evaluation, we preserved the default input tensor size in all training (640 × 640 × 3), setting to zero the unused channels for each experiment. We also evaluated the 5 pre-defined segmentation models with different sizes that are available in the YOLOv8 official repository [49]: Nano (N), Small (S), Medium (M), Large (L), and eXtra-large (X).

For each possible combination of input channels (7) and CNN sizes (5), we ran a dedicated training of 250 epochs on a Nvidia GeForce RTX 3090 GPU. We used image data augmentation operations during all training sessions: horizontal flipping, HSV color variation, translation, scaling, and mosaic composition.

We compared our results with other state-of-the-art techniques (SECOND [30], CenterPoint [33], PointPillars [51], PointRCNN [16] and PV-RCNN [52]), trained with the same data we used for our models, for the same number of epochs and evaluated over the same test acquisitions.

The metric used for the evaluation is the average precision (AP) with an intersection over union (IoU) threshold of 50% (AP_50_), 75% (AP_75_) and the mean average precision for IoU thresholds between 50% and 95% with steps of 5% (mAP_50:95_), estimated by sampling the precision–recall (PR) curve with 40 points, as detailed in [53].

**Figure 5:**
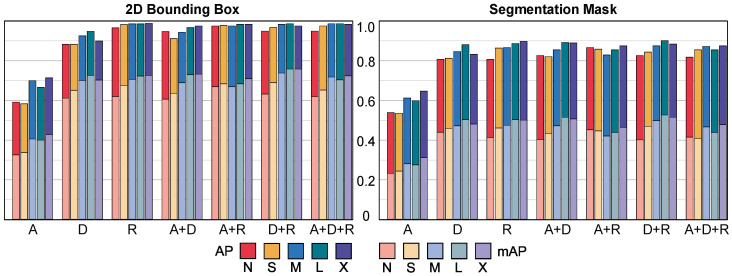
Ablation test results. CNN performance without post-processing for each CNN size nano, small, medium, large, extra-large) and each possible combination of input channels, (ambient, depth, reflectivity, A+D, A+R, D+R, A+D+R).

**Figure 6:**
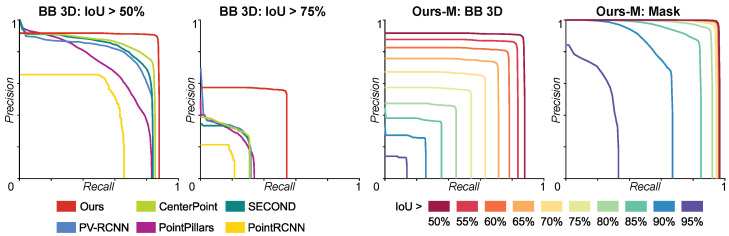
Full pipeline performance, using the Medium (M) CNN and with all input channels (A+D+R). PR curves and comparison with other techniques when predicting 3D bounding boxes with IoU thresholds of 50% (first plot) and 75% (second plot). PR curves for different IoU thresholds when predicting 3D bounding boxes (third plot) and segmentation masks (fourth plot).

Figure 5 shows the AP_50_ and mAP_50:95_ of the trained CNNs without post-processing for different combinations of input channels and CNN sizes. We report the performance of the estimated 2D bounding boxes (left) and the segmentation masks (right). Figure 6 shows the results of our full pipeline using all input channels (A+D+R) with the Medium (M) model size and how it compares to the other techniques when predicting 3D bounding boxes (first two plots). It also shows the precision–recall curves at different IoU thresholds for 3D bounding boxes (third plot) and for segmentation masks (fourth plot). Table 2 compares our full pipeline (considering all input channels and different CNN sizes) with the other techniques when predicting 3D bounding boxes in terms of AP_50_ score, AP_75_ score, mAP_50:95_ score and average execution time per scan. Finally, Table 3 shows the performance of our full pipeline (AP_50_, AP_75_ and mAP_50:95_) for segmentation masks for different CNN sizes and considering all input channels.

As Figure 5 shows, the average performance increases with the size of the model. The only exception is the extra-large (X) model, which probably required more training to reach its maximum performance. However, together with performance, inference time and memory requirements also increase, as shown in the last column of Table 2.

The ambient light channel is the one that contributes the least to the overall performance. In general terms, reflectivity seems to be the most contributing one. However, with high overlaps (mAP_50:95_), the range component provides similar or even better results. The combination of both is, on average, the one that provides the highest scores. This could be explained by the fact that most of the data were acquired indoors, where ambient light is low. As Figure 1 shows, the ambient channel is noisy in such conditions. Also, both depth and reflectivity are *active* channels, i.e., do not depend on the ambient illumination, which makes them more attractive from a robustness point of view.

In our evaluation, the post-processing stage filtered out, on average, 11% of the points originally selected by the CNN, improving the overall mask IoU from 88.63% to 94.54%. Its individual contribution can be analyzed by comparing the results of Figure 5-right (segmentation masks using only the CNN) and Table 3 (segmentation masks using the full pipeline): enabling the post-processing stage improved the AP_50_ by 8.98% (from 85.62% to 94.60%) and the mAP_50:95_ by 37.03% (from 44.09% to 81.12%).

The last column of Table 3 shows the execution time of our full pipeline. This time is the same for both cases, 3D bounding boxes (Table 2) and segmentation masks (Table 3). It accounts for both the CNN inference time and the post-processing time (which was, on average, 2.27 ms). Considering that the sensor can provide scans at a maximum frequency of 20 Hz and the times reported in Table 3, real-time capabilities are always ensured, with speeds that range between 201 scans per second for the *N* model to 50 scans per second for the *X* one.

The results for the other techniques shown in Table 2 after training/evaluating them in our dataset are better than the ones officially reported with the KITTI dataset [8]. This can be explained by the fact that our sensor, compared with the one used in the KITTI dataset, provides twice the vertical resolution (128 lines compared to 64), which simplifies the problem. The only exception is PointRCNN: since it imposes a limit on the number of input points, higher-resolution point clouds do not necessarily yield better results. Overall, any of our models outperform the others when estimating 3D bounding boxes in terms of average precision, with lower inference times that enable real-time applications.

It is important to remark that 3D bounding boxes are not natively provided by our technique: the CNN training is performed in 2D and, consequently, the inference results are 2D bounding boxes and segmentation masks. Unlike segmentation masks, which can be directly applied to the 3D points, 2D bounding boxes cannot be directly converted into 3D bounding boxes. To perform the comparison shown in Table 2, we indirectly estimated the 3D bounding boxes using the points selected by the segmentation masks and computing the minimal axis-aligned 3D bounding box that contains them. In this sense, a single miss-classified point can randomly increase the volume of the bounding box and have a critical impact on the final performance. As shown in Figure 6, the PR curve for our technique with an IoU greater than 50% (first plot) is only relatively better than the ones from the other techniques, as several bounding boxes failed the intersection test due to a few miss-classified background points. However, as the IoU threshold increases (second and third plots), the remaining boxes with no outliers are more resilient than the ones produced by the other techniques, providing better AP_75_ and mAP_50:95_ scores.

The inference of segmentation masks improves notably after including the post-processing stage. In principle, segmentation constitutes a more complex and computationally demanding problem than just detection. However, with our technique, we achieve much better results in this task without an impact on the execution time. As Table 3 and Figure 6-right show, the AP_50_ scores are always above 90%, and the mAP_50:95_ scores are around 80%, with only the *nano* model below this mark. When compared with the raw inferences provided by the model (Figure 5-right), the improvement is significant. In Figure 6-right, it can be observed how PR curves remain practically identical for IoU thresholds between 50% and 70%. It is only from thresholds above 75% where a slight decay in performance can be observed and only above 85% where results clearly degrade.

## 4. Discussion

We have presented an approach that benefits from both the unique features of lasers and the state of maturity of DL models for 2D visual object recognition.

We have introduced a novel pipeline that projects structured LiDAR data into the standard input of 2D models without loss of information. The outcome is an end-to-end 3D object recognition technique that overcomes the main differences between cameras and lasers and produces consistent 3D results. To the best of our knowledge, this is the first work that faces this problem without explicitly re-projecting point clouds and, consequently, without suffering from the associated data degradation and computational overhead. Moreover, this is the first technique that exploits, simultaneously, the main three channels that modern laser sensors provide.

Given the novel way in which our approach treats laser data (both in terms of structure and channels), we could not benefit from already existing datasets for training/evaluating our technique. We have introduced a novel 3D data-mining technique that can be easily generalized to unstructured sensors. It exploits the capabilities of laser sensors to robustly perform background subtraction operations in order to automatically annotate 3D scans. The training and evaluation of our pipeline have been entirely performed over the results of this technique. Additionally, together with this paper, we have presented a new public dataset that includes all the annotated data that we have produced.

Our technique outperforms the state-of-the-art models in detection tasks. However, it does not provide 3D bounding boxes natively; they are indirectly estimated from segmentation masks. As the results show, precision scores for segmentation masks are notably higher than just for bounding boxes, which typically pose a simpler problem and offer a less informative output. On top of that, inference time is significantly reduced, enabling real-time applications.

The results of the ablation analysis have shown the benefits of *native* laser data, where the combination of the two active channels (range and reflectivity) have provided the highest precision values. This eliminates the dependency on controlled ambient light and expands the application fields of the technique. To the best of our knowledge, this is the first time that such ablation analysis has been performed.

Future works may assess more in depth the impact of the post-processing stage in the final accuracy results. This analysis could provide valuable insights into the specific effects of the shrink operation and contribute to a more comprehensive understanding of its role in the overall methodology.

## Figures and Tables

**Table 1:** Dataset details. For each acquisition, the number of background scans, i.e., no instances, foreground scans, i.e., at least one instance, the number of frames wrongly annotated by our automatic technique, and the total number of instances.

Acquisition	Background	Foreground	Invalid	Masks
coffee1	60	1137	47	3487
coffee2	63	1134	19	1952
corridor1	101	1096	51	1430
corridor2	91	1106	15	1903
lab1	88	1109	21	1088
lab2	68	1129	65	1066
lab3	37	1160	9	1151
lab4	88	1109	31	1951
lab5	60	1137	68	3561
out1	54	1143	5	4329
out2	34	1163	3	3998
out3	42	1155	0	2282
**Total**	**786**	**13,578**	**334**	**28,198**

**Table 2:** 3D Bounding Box prediction results. AP_50_ (first column), AP_75_ (second column), mAP_50:95_ (third column) scores and inference time in milliseconds (fourth column) for our full pipeline with all channels (A+D+R) and different backbone sizes (first 5 rows) and for other state-of-the-art techniques.

	AP_50_	AP_75_	mAP	Time
Ours-N	72.36%	24.81%	33.02%	**4.97**
Ours-S	75.97%	27.56%	35.57%	5.93
Ours-M	**79.01%**	29.92%	38.03%	9.88
Ours-L	78.25%	**31.40%**	**38.10%**	13.87
Ours-X	75.81%	30.83%	37.01%	19.94
CenterPoint [33]	73.12%	9.93%	24.54%	96.97
SECOND [30]	70.15%	9.45%	23.53%	65.12
PV-RCNN [52]	67.59%	9.90%	22.94%	195.3
PointPillars [51]	60.41%	9.74%	21.17%	63.02
PointRCNN [16]	40.25%	4.11%	11.67%	118.7

**Table 3:** Segmentation mask results for our full pipeline with all channels (A+D+R) and different backbone sizes. AP_50_ (first column), AP_75_ (second column), mAP_50:95_ (third column) scores and inference time in milliseconds (fourth column).

	AP_50_	AP_75_	mAP	Time
Ours-N	93.67%	90.79%	79.34%	**4.97**
Ours-S	94.68%	91.91%	80.77%	5.93
Ours-M	94.93%	92.13%	81.58%	9.88
Ours-L	**94.94%**	**93.74%**	**82.41%**	13.87
Ours-X	94.72%	92.02%	81.52%	19.94

## Data Availability

The original data presented in the study are openly available in [50].
